# Influence of Follicular Fluid and Seminal Plasma on The Expression of Endometrial Receptivity Genes in Endometrial Cells

**DOI:** 10.22074/cellj.2021.6851

**Published:** 2020-04-22

**Authors:** Tamouchin Moharrami, Jafar Ai, Somayeh Ebrahimi-Barough, Mohammad Nouri, Maryam Ziadi, Hossein Pashaiefar, Yazarlou Fatemeh, Mohammad Ahmadvand, Soheil Najafi, Mohammad Hossein Modarressi

**Affiliations:** 1. Department of Medical Genetics, Tehran University of Medical Sciences, Tehran, Iran; 2. Department of Tissue Engineering and Applied Cell Sciences, Faculty of Advanced Technologies in Medicine, Tehran University of Medical Sciences, Tehran, Iran; 3. Stem Cell Research Center, Tabriz University of Medical Sciences, Tabriz, Iran; 4. Department of Medical Genetics, Tabriz University of Medical Sciences, Tabriz, Iran; 5. Hematology, Oncology and Stem Cell Transplantation Research Center, Tehran University of Medical Sciences, Tehran, Iran; 6. Department of Immunology, School of Public Health, Tehran University of Medical Sciences, Tehran, Iran

**Keywords:** Endometrium, Follicular Fluid, Implantation, Seminal Plasma

## Abstract

**Objective:**

Endometrial receptivity plays a key role in pregnancy success in assisted reproduction cycles. Recent evidence suggests that seminal plasma (SP) and follicular fluid (FF) influence the uterine endometrium to improve implantation of the embryo and the establishment of pregnancy. In this study, we attempt to assess the influence of FF and SP on the expression levels of main endometrial receptivity genes (*HOXA10, HOXA11, ITGAV, ITGB3* and *LIF*) in endometrial stromal cells.

**Materials and Methods:**

In this experimental study, SP and FF were collected from 15 healthy fertile men and 15 healthy fertile women, respectively. Tissue specimens of the endometrium were obtained from 12 women undergoing hysterectomy for benign conditions. After endometrial stromal cell isolation and culture, dose- and time-dependent cytotoxic effects of pooled FF and SP on 3D-cultured endometrial cells were evaluated. A second independent set of 12 endometrium samples was treated under determined optimum conditions and evaluated for gene expression analysis using quantitative real-time polymerase chain reaction (qRT–PCR).

**Results:**

The results of this study indicated that exposure of endometrial stromal cells to FF resulted in the elevated expression of *HOXA10* (fold change=2.6, P=0.02), *HOXA11* (fold change=3.3, P=0.002), *LIF* (fold change=4.6, P=0.0003), *ITGB3* (fold change=3.5, P=0.012), and *ITGAV* (fold change=2.8, P=0.001) compared to untreated cells. In addition, we found that SP-treated endometrial cells showed increased mRNA levels of only the *LIF* gene (fold change=2.5, P=0.008) compared to untreated cells.

**Conclusion:**

Human SP and FF may modulate the endometrial receptivity and improve the implantation rate in assisted reproduction cycles through the up-regulation of endometrial receptivity genes.

## Introduction

Assisted reproductive technologies (ART) are not only used to overcome fertility issues in infertile cases but are also key tools in preventing genetic abnormalities in fertile couples. However, the efficacy of ARTs is still suboptimal and the low rates of transferred embryo implantation in ART cycles remains the main challenge for achieving successful pregnancies. This limitation partly results from inadequate knowledge about the cellular and molecular basis of germ cells, embryos, and endometrium physiology (1). Despite recent advances in embryo development, selection, and transfer techniques, implantation failure occurring in approximately 75% of cases is a major limiting factor for pregnancy following *in vitro* fertilization (IVF) attempts (2). The receptive endometrium is one of the most important factors for the outcome of pregnancies following ART cycles and optimizing endometrial receptivity is imperative to improving the success rate of ART. During the implantation window, a unique timeframe in which implantation is possible, the endometrium plays a crucial role in successful implantation (3).

Endometrial receptivity and subsequent embryo implantation can only happen after a complex series of histological, cellular and molecular changes in the endometrium. It has been shown that successful embryo implantation depends on an ideal endometrium-embryo cross-talk through the known crucial growth factors and cytokines which are secreted from endometrial cells (4).

Previous studies have indicated that differential expression of a variety of genes including those involved in immune response, the complement cascade pathway, cell adhesion and exosome biogenesis may influence endometrial receptivity (5). Hence, in the past decade, many global transcriptomic studies have been designed to find potential biomarkers or molecular signatures for a receptive endometrium. There are, however, many disagreeing reports due to differences in the analysis of gene expression and sample selection (6, 7). Moreover, different studies have suggested that the use of supplements such as vitamins, hormones and minerals may improve endometrial receptivity and increase the chance of implantation in ART procedures (8, 9) .

Due to the complex nature of endometrial receptivity, it seems the use of a cocktail of supplements can be more effective than individual supplements. Seminal plasma (SP) and Follicular fluid (FF) can be considered as cocktails of various natural biocompounds. SP is a reach medium comprised of different biologically active factors including cytokines, chemokines, prostaglandins, growth factors, angiogenic factors, vitamins, zinc, etc (10). According to recent proteomic studies, high concentrations of these cytokines and prostaglandins in the SP of fertile men and aberrant concentrations of these components in the SP of infertile men demonstrate that SP constituents may play a key role in human reproduction. Furthermore, most of these important compounds which have been identified in the female reproductive tract (FRT) suggest their involvement in the regulation of FRT functions and successful reproduction (11). From the mechanistic point of view, different studies in mice, pigs and humans have shown that SP may play a critical role in endometrial receptivity and the improvement of implantation chances (12, 13). In addition to SP, FF which provides critical factors for oocyte development, contains important cytokines, hormones and growth factors that may mediate paracrine/autocrine interactions during the process of implantation (14).

During ART cycles such as IVF or ICSI, unlike what happens naturally in the body of mammals, the blastocyst is transferred without any SP and the FF is also discarded during ovum pick-up. As these fluids contain a vast range of natural elements including growth factors that possibly improve receptivity of the endometrium, we hypothesize that using the blastocyst alone might cause limited implantation rates after ART cycles. We evaluated this hypothesis through the study of the *in vitro* influence of SP and FF on the expression levels of the genes *HOXA10, HOXA11, LIF, ITGB3,* and *ITGAV*, whose role in the successful implantation of blastocysts have been established, in endometrial stromal cells.

## Materials and Methods

### Endometrial tissue collection and processing

In this experimental study, twelve fresh endometrial tissue samples were collected from childbearing-age women (ages 23-35) undergoing hysterectomy for benign conditions. Six of the participants were receiving hysterectomies for fibroids, three for adenomyosis, one for uterine prolapse and one for heavy menstrual bleeding. All of them were in the secretory phase of their menstrual cycle. Malignancy, drug and hormone therapy, and pregnancy were the exclusion criteria for the participants in our study. Written informed consent was obtained from all participants and the study was approved by the Ethics Committee of Tehran University of Medical Sciences (IR. TUMS.MEDICINE.REC.1396.4258). Immediately after sampling, the endometrium specimens were placed in Hank’s Balanced Salt Solution (HBSS, Sigma-Aldrich, USA) with 1% penicillin/streptomycin (Pen/Strep, Gibco, USA) and transported to the laboratory within two hours. Tissue samples were then transferred to sterile 10 cm petri dishes and rinsed with phosphate buffered saline (PBS, Merck, Germany). After washing, the tissue samples were transferred to another petri dish containing pre-warmed HBSS and were cut into small pieces with a sterile scalpel. The dissected specimens were transferred into sterile 15 ml tubes containing HBSS and 3 mg/ml collagenase type I (Sigma-Aldrich, USA) for enzymatic digestion. After 60 minutes of incubation at 37˚C, the solutions were resuspended in Dulbecco’s Modified Eagle Medium and Ham’s F-12 (DMEM/F12, Gibco, USA) supplemented with 10% fetal bovine serum (FBS, Gibco, USA) for enzyme neutralization. Following complete tissue digestion, cell suspensions were filtered through 70 micron cell strainers to remove the undigested fragments from the suspension. Afterwards, the cell suspensions were filtered through a 40 micron cell strainer to isolate the endometrial stromal cells from endometrial epithelial cells.

For further purification and removal of red blood cells (RBCs), fresh DMEM/F12 culture medium supplemented with 10% FBS was added to the collected cells and centrifuged at 1500 rpm for 10 minutes. After removing the supernatant, the cell pellet was resuspended and added to Ficoll-Paque media solution in 15 ml tubes, then centrifuged for 20 minutes at 1200 rpm. Afterward, the upper layer containing stromal cells was transferred to a new tube and washed twice with PBS (15) including osteocytes and adipocytes. Here, the potency of EnSC in neural differentiation has been investigated. Flow cytometric analysis showed that they were positive for CD90, CD105, OCT4, CD44 and negative for CD31, CD34, CD133. The characterized cells were induced into neural differentiation by bFGF (basic fibroblast growth factor

### Primary cell cultures

The stromal cells were transferred to T_25_ culture flasks containing DMEM/F12, 10% FBS and 1% Pen/Strep and were incubated at 37˚C and 5% CO_2_. After 24 hours of incubation, the culture medium and nonadherent cells were discarded and the attached cells were washed twice with PBS, then fresh culture medium was added and incubated at 37˚C and 5% CO_2_. The medium was changed every 3 days until passage 3. Cells of passage 3 were used for the experiments

### Three-dimensional cell culture

Fibrin gel was used to provide a three-dimensional matrix for culturing endometrial stromal cells. The 3D cultures of for MTT (3-(4,5-Dimethylthiazol-2-yl)- 2,5-diphenyltetrazolium bromide) assays were carried out in 96-well culture plates while other cultures took place in 24-well plates for optimized treatment. Fibrin gel, were produced by dissolving 3 mg of fibrinogen (Sigma, USA) in 1 ml of M199 medium (Sigma, USA). Stromal cells (2×10^5^ cells/ml) were added to the prepared fibrinogen solution and carefully mixed with 2 μl of a thrombin solution (120 U/ml in 1 M sodium buffer, Sigma, USA), 1.5 μl of CaCl_2_ (1%). Then 100μl of cell containing fibrinogen medium was added to each well of a 96-well cell culture plate. The plate was incubated at 37˚C for 1-2 hours to form a threedimensional structure. Following fibrin gel formation, 0.1 ml of DMEM/F12 medium supplemented with 10% FBS was added to each well and the plate was returned to the 37˚C incubator. The culture medium was refreshed every 3 days following a previously published protocol (16).

### Seminal plasma and follicular fluid preparation

Semen samples were collected from 15 fertile donors, aged 27-41 years old (mean age of 34), with normal spermogram from the Reproductive Health Center of Tabriz Alzahra Hospital. The samples were centrifuged at 3000 rpm for 20 minutes. Supernatants were collected and centrifuged again at 10000 rpm for 15 minutes in order to remove. The supernatants were pooled, filtered through 0.22 μm filters for the prevention of microbial contamination and stored at -20˚C until used.

FF was obtained by puncturing ovarian follicles from 15 ovum donors, aged 23-32 (mean age of 27), at Reproductive Health Center of Tabriz Alzahra Hospital. Macroscopically clear FF samples were centrifuged at 6000 rpm for 20 minutes to remove cellular components. The supernatants were pooled, filtered through 0.22 μm filters and stored at -20˚C until used.

### Seminal plasma and follicular fluid toxicity evaluation using the MTT assay

The MTT assay was used to determine the nontoxic doses and effects of semen plasma and FF on viability of the fibrin gel-encapsulated endometrial stromal cells. The MTT assay is a reliable colorimetric reaction, which is widely used to measure cell viability and cytotoxicity. The principle of this assay is the reduction of MTT dye to formazan crystals by the mitochondrial dehydrogenases of viable cells. There is a linear correlation between the amount of formazan and cell viability. So, determination of formazan quantity can be used as an estimate of the population of living cells.

A total of 10^4^ endometrial stromal cells isolated from three different samples were encapsulated in fibrin gel (3 mg/ml) in separate wells of 96-well plate. Different concentrations of FF and semen plasma (1%, 5%, 10%, 20%, 50% and 100%) were added to each well as treatment groups. These concentrations were achieved through dilution with DMEM/F12 culture medium containing 2% FBS. The control group was endometrial stromal cells encapsulated in fibrin gel without any treatments.

After treatment with SP for 3, 6, 24, 48 and 72 hours or FF for 3, 6, 24, 48 72, and 96 hours, the culture media were removed and 100 µl of MTT solution (0.5 mg/ml in PBS, Sigma, USA) was added to each well and incubated at 37˚C for 4 hours. Afterward, 100 µl of dimethyl sulfoxide (DMSO, Sigma Aldrich, USA) was added to each well and incubated for 15 minutes in a dark room at room temperature to dissolve the formazan crystals. Finally, absorbance was measured at 570 nm using a spectrophotometric plate reader Asys Expert 96 (Biochrom, UK) according to established guidelines. These experiments were repeated five times. Cell survival was calculated as the percentage of test absorbance compared to the control absorbance.

### RNA isolation and quantitative real-time polymerase chain reaction

Total RNA was extracted from cultured endometrial stromal cells using TriPure Isolation Reagent (Roche, Switzerland) according to the manufacturer’s instructions. Concentration and purity of extracted RNA samples were checked with a NanoDrop 2000C spectrometer (Thermo Scientific, USA) and RNA integrity was evaluated by running 1 µl of total RNA samples through 1% agarose gel. A total 1 μg of extracted RNA was retrotranscribed to complementary DNA (cDNA) with random hexamer and oligo dT primers, using a cDNA synthesis kit (Takara, Japan). The final cDNA product was used as the template for qRT-PCR.

Quantitative real-time PCR was done in duplicates on a light cycler 96 real-time PCR system (Roche, Switzerland). The qRT-PCR reactions were run with the following settings: 95˚C for 10 minutes, followed by 45 cycles of 95˚C for 10 seconds and 60˚C for the 30 seconds. At the end of each qRT-PCR run, the melting curve program was run to make sure the PCR product’s specificity. Each qRT-PCR reaction contained 10 µl RealQ Plus Master Mix Green (Ampliqon- Denmark), 1 µl (100 ng/µl) of cDNA, 1µl mixed forward and reverse primers and 8 µl ddH_2_O. Expression levels of the target genes were evaluated by normalizing to the expression of the *GAPDH* gene as the reference gene. The relative expression levels of target transcripts were calculated through the 2^−ΔΔCt^ method. Primer sequences and characteristics are summarized in Table 1.

**Table 1:** Primer sequences used for quantitative real-time polymerase chain reaction


Gene name	Transcript	Primer sequence (5ˊ-3ˊ)	Annealing T_m_(˚C)	Amplicon size (bp)

*HOXA10*	NM_018951.4	F: GGATTCCCTGGGCAATTCCA	60	99
		R: AGTGTCTGGTGCTTCGTGTA		
*HOXA11*	NM_005523.5	F: CCAGAATGAGGCTGCTTTCC	59	173
		R: GAACTCAGGGCTGGATCAGT		
*ITGAV*	NM_002210.5	F: TGGAGCACCTCTCTTCATGG	60	177
		R: CCATCCTGGTCCAGATCTCC		
*ITGB3*	NM_000212.2	F: CTCCTCATCACCATCCACGA	59	84
		R: GTTGTTGGCTGTGTCCCATT		
*LIF*	NM_002309.5	R: ACATCTGGACCCAACTCCTG	59	131
		F: AGAAGAAGAAGCTGGGCTGT		
*GAPDH*	NM_002046	F: GAAGGTGAAGGTCGGAGTCA	60	109
		R: ATTGAAGGGGTCATTGATGG		


### Statistical analysis

The obtained data were analyzed statistically using the SPSS 20.0 software package (SPSS, Chicago, IL, USA). Paired t test was applied to assess statistical significance differences as appropriate. For all statistical analyses, a P<0.05 was considered to be statistically significant.

## Results

### Cytotoxicity assays

Before analyzing of the effects of semen plasma and FF on the expression levels of receptivity genes in endometrial stromal cells, we determined the optimum FF and SP concentrations and treatment time that had no dose- or time-dependent cytotoxic effects on the 3D- cultured endometrial stromal cells obtained from 3 different women.

**Fig.1:**
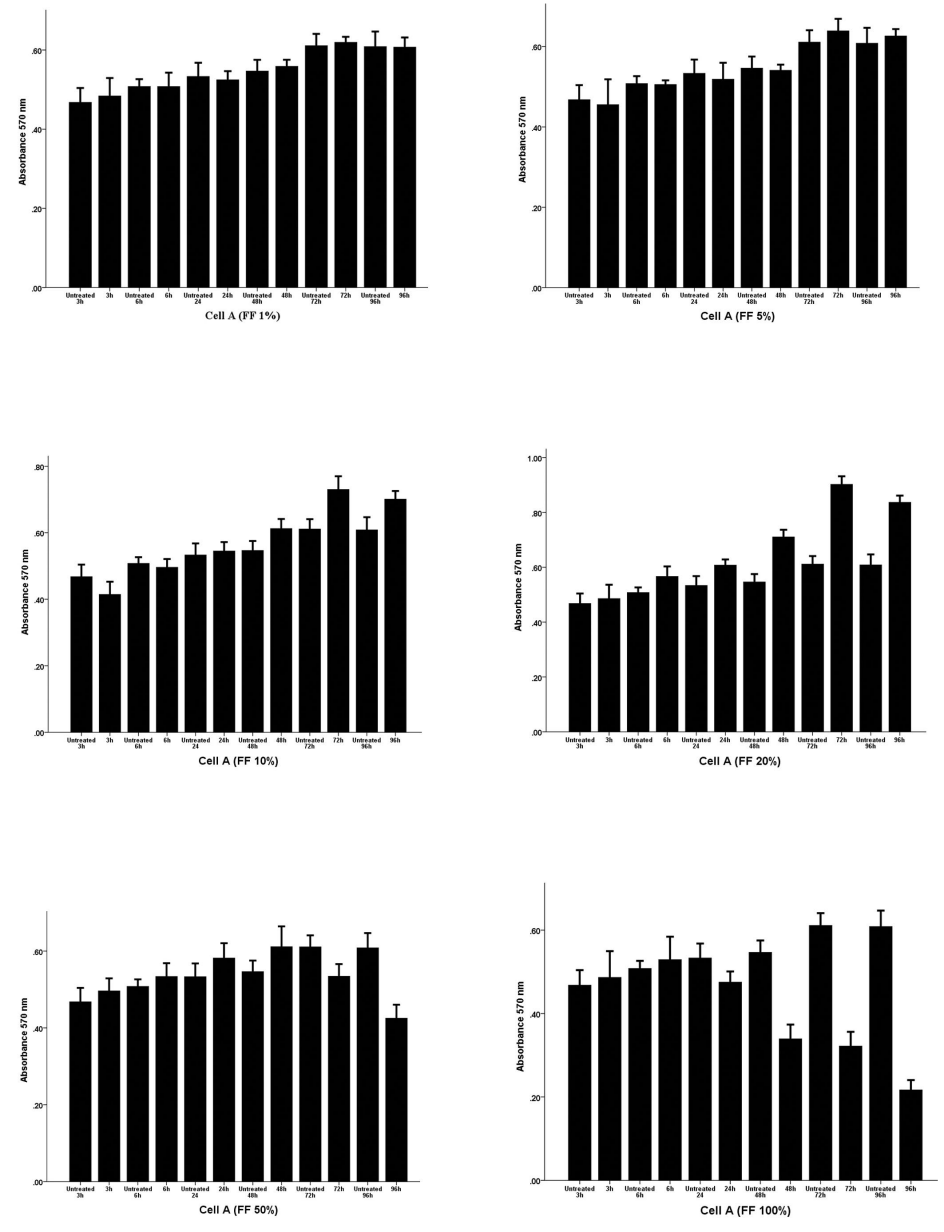
Analysis of the dose- and time-dependent effect of follicular fluid (FF) on the viability of endometrial stromal cells using the MTT assay. The endometrial cells were exposed to different concentrations of FF (0, 1, 5, 10, 20, 50 and 100%) for 3, 6, 24, 48, 72 and 96 hours. The numbers on the bars show mean absorbance at 570 nm.

**Fig.2:**
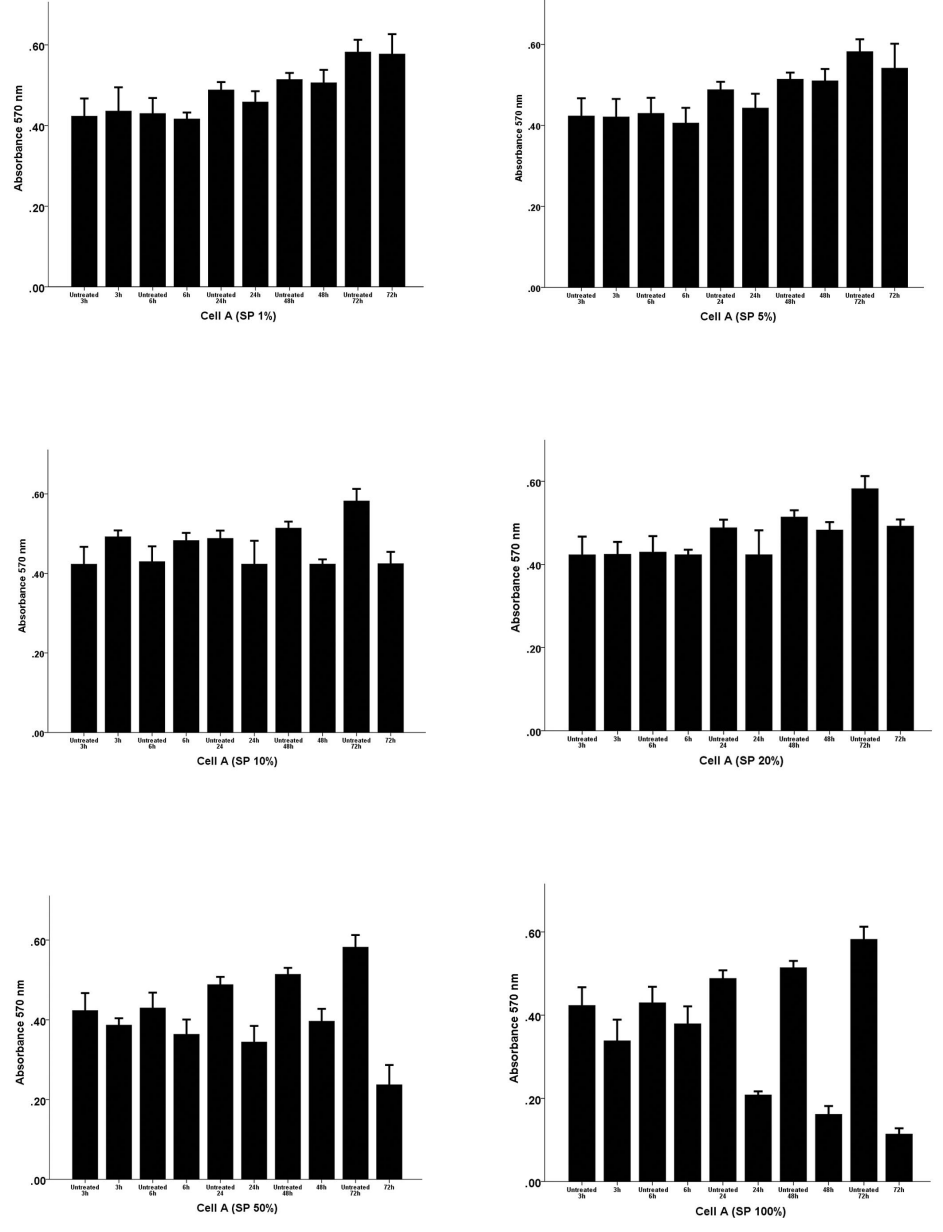
Analysis of the dose- and time-dependent effect of follicular fluid (FF) on the viability of endometrial stromal cells using the MTT assay. The endometrial cells were exposed to different concentrations of FF (0, 1, 5, 10, 20, 50 and 100%) for 3, 6, 24, 48, 72 and 96 hours. The numbers on the bars show mean absorbance at 570 nm.

As shown in Figures 1 and 2, respectively, we found that a 72-hour incubation with 20% FF and a 48-hour incubation with 10% SP resulted in the highest proliferation rate and had no cytotoxic effects on the cultured cells. Therefore, these non-toxic conditions were chosen for subsequent qRT-PCR analyses.

### Influence of follicular fluid and seminal plasma on the expression of endometrial receptivity genes

To evaluate the capacity of SP and FF, to modulate expression of endometrial receptivity genes, cultured endometrial stromal cells were exposed to either vehicle control medium alone, 10% SP for 48 hours or 20% FF for 72 hours. mRNA expression levels of *LIF, ITGB3, ITGAV, HOXA11,* and *HOXA10* were analyzed with qRTPCR.

**Fig.3:**
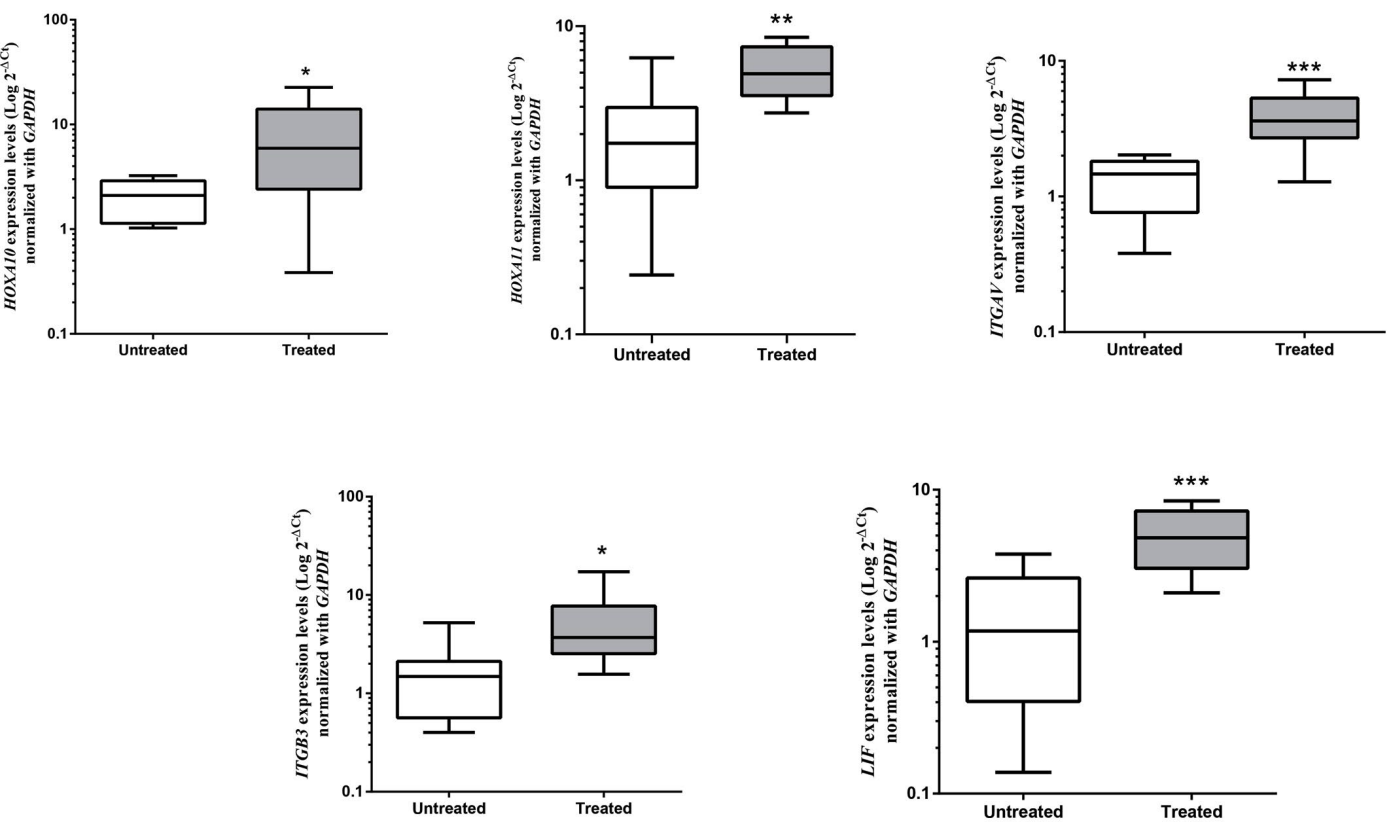
The relative mRNA expression levels of endometrial receptivity genes following follicular fluid (FF) treatment. The line in each box indicates the mean expression level and bars represent confidence interval (CI) 95%. Gene expression levels are indicated in Log scales. *; P≤0.05, **; P≤0.01, and ***; P≤0.001.

The exposure of endometrial stromal cells to FF resulted in elevated expression of *HOXA10* (fold change=2.6, P=0.02), *HOXA11* (fold change=3.3, P=0.002), *LIF* (fold change=4.6, P=0.0003), ITGB3 (fold change=3.5, P=0.012) and *ITGAV* (fold change=2.8, P=0.001) compared to vehicle control medium alone (Fig.3).

**Fig.4:**
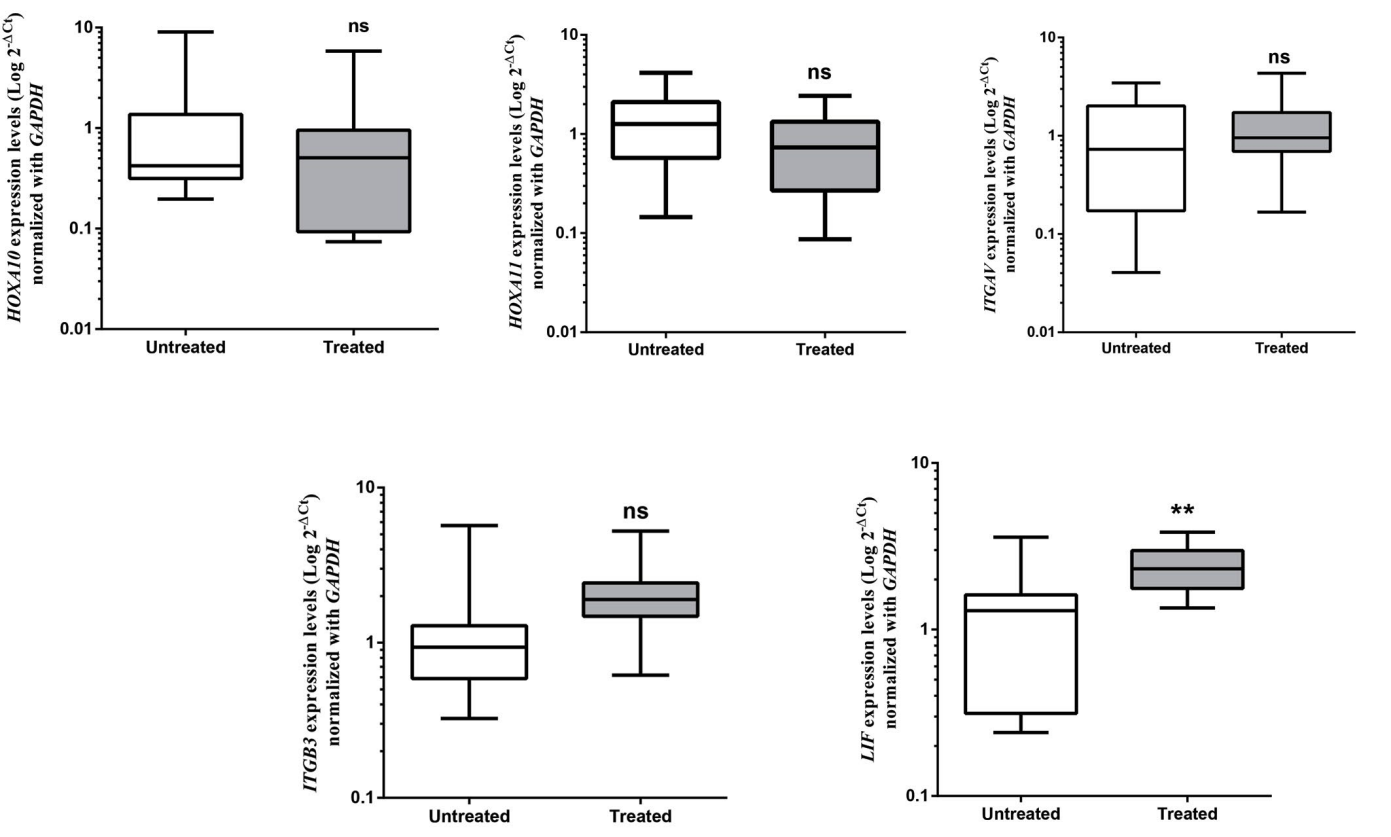
The relative mRNA expression levels of endometrial receptivity genes following follicular fluid (FF) treatment. The line in each box indicates the mean expression level and bars represent confidence interval (CI) 95%. Gene expression levels are indicated in Log scales. *; P≤0.05, **; P≤0.01, and ***; P≤0.001.

In addition, we found that in SP treated endometrial stromal cells, only the mRNA levels of the *LIF* gene was increased (fold change=2.5, P=0.008) compared with vehicle control medium while the expression of *ITGB3, ITGAV, HOXA11,* and *HOXA10* did not change (Fig.4).

## Discussion

During the past two decades, ART has become a key medical procedure to help infertile women achieve pregnancy. However, embryo implantation failure in ART cycles remains the main obstacle for achieving a successful pregnancy. The implantation of the blastocyst is a complex process involving reciprocal communication between the embryo and the uterus, which is mainly dependent on the function and receptivity of the endometrium. It is thought that suboptimal endometrial receptivity is the reason for two-thirds of implantation failures. Thus, different clinical strategies have been employed to improve implantation rates following ART cycles (17).

Many different histological, biochemical, and molecular genetics studies have been conducted with the aim of defining endometrial receptivity markers. Identification of these markers can be very useful in using them in diagnostic setting and improving ART methods. Of these markers, molecular markers are of a great importance due to their high sensitivity and specificity (7). That is why many transcriptomic and proteomic investigations have been performed in the past decade to find reliable molecular markers that reflect the level of the endometrium’s receptivity (18). Discovery of these markers and the key molecular pathways involved in the implantation process can facilitate improvement of the endometrium receptivity methods (19). However, due to considerable differences among the results of these studies, there is no consensus on the genes that can be used as biomarkers in clinical diagnostic tests for determining of the level of endometrial receptivity (7). Nevertheless, the central roles of some gene families and signaling pathways that are involved in implantation have been established in endometrial cells. These gene families are mainly growth factors, cytokines, chemokines and cell adhesion molecules. The fact that a variety of molecular mechanisms such as disrupted growth factor, cytokine, and hormone signaling are thought to be involved in the suppression of endometrial receptivity biomolecules that lead to a reduced implantation rate further confirms this (20).

It therefore seems that one main approach for enhancing endometrial receptivity can be exposure of the endometrium to a biological cocktail containing various biologically active factors. It is believed that human semen plasma and FF naturally contain the critical signaling components that have major functions in the process of implantation. Furthermore, recent studies have indicated that SP and FF which can enter the uterine cavity during sexual intercourse ovulation respectively, may affect and regulate endometrial signaling mechanisms to induce implantation and increase reproductive success (21).

Several studies have assessed the *in vitro* and *in vivo* effects of SP and FF on the functions and gene expression of the endometrium. Chen et al. (22) examined the *in vitro* effects of SP on the transcriptome of human endometrial cells. Their results indicated that SP exposure leads to upregulation of genes involved in proliferation, viability, and migration in the endometrial stromal cells. Gutsche et al. (23) indicated that SP has an *in vitro* stimulatory effect on the expression levels of pro-inflammatory cytokines including IL-1b, IL-6 and, LIF in human endometrial cells. In agreement with the previous studies, a recent study by Rodriguez-Caro et al. (24) has proved that *in vitro* interaction between SP extracellular vesicles and endometrial stromal cells may improve endometrial receptivity by inducing prolactin secretion, which is a key hormone in implantation, and enhancing decidualization. A recent systematic review and meta-analysis conducted by Saccone et al. (11) indicated a higher clinical pregnancy rate after intra-vaginal/cervical injection of SP at the time of oocyte pickup suggesting that SP plays an important role on endometrial function and the maternal immune system, and thereby supports implantation. A prospective randomized study by Hashish et al. (25) showed that flushing of the endometrial cavity with FF in patients undergoing intracytoplasmic sperm injection (ICSI) did not significantly improve implantation rates. However, FF is rich in growth factors and its potential for increasing the rate of implantation and subsequent successful pregnancy should be the focus of further research.

To address these challenges, in the current study, we determined the *in vitro* influence of FF and SP on the expression levels of main endometrial receptivity genes with established roles in the implantation process including *HOXA10, HOXA11, ITGAV, ITGB3* and *LIF*, in endometrial stromal cells

We have applied a fibrin three-dimensional cell culture system for endometrial stromal cells culture to provide information that is more physiologically relevant to cells *in vivo*. This polymer has recently become widely used in tissue engineering. Fibrin gel is naturally formed in the body, through the natural process of fibrinogen mixing with thrombin (26).

We first ensured that the response to FF or SP was not due to their cytotoxic effects by performing precise cytotoxicity assays. We found that a 48 hours incubation with 10% SP and a 72 hours incubation with 20% FF showed they had no dose- or time-dependent cytotoxic effects on the endometrial stromal cells and interestingly, the maximum viability for cells was seen in these conditions.

Exposure of endometrial stromal cells to FF resulted in elevated expression of all of the analyzed genes including an increase of 2.6 fold in *HOXA10*, 3.3 fold in *HOXA11*, 4.6 fold in *LIF*, 3.5 fold in *ITGB3* and 2.8 fold in *ITGAV* expression levels. In addition, we found that SP-treated endometrial cells only showed an increase of 2.5 fold in the mRNA levels of the *LIF* gene compared to untreated controls.

In this study, we selected genes whose essential role in fetus implantation has been proven by different studies, and are involved in the key signaling pathways. *HOXA10* and *HOXA11* are two important members of the homeobox gene family, and encode very important transcription factors. It has been proven that expression of *HOXA10*, a member of a family of homeobox genes, is critical for FRT development and endometrial receptivity. Moreover, up-regulation of *HOXA10* at the time of implantation improves endometrial receptivity by regulating downstream genes such as ITGB3 (27). A supporting study, Wang et al. (28) reported that 5-Aza- 20-deoxycytidine (AZA) might improve endometrial receptivity through the induction of *HOXA10* expression.

Like *HOXA10, HOXA11* expression plays an important role in implantation and the down regulation of this gene leads to female infertility. Therefore, upregulation of both *HOXA10* and *HOXA11* in the receptive endometrium indicate that these genes play important roles in decidualization. Women with abnormal expression of *HOXA10* and *HOXA11* genes show lower rates of implantation indicating that these genes are important for blastocyst implantation because they regulate the expression of molecular and cellular markers needed for embryo implantation (29).

*ITGAV* and *ITGB3* encode essential cell adhesion molecules. *ITGAV*, a member of the integrin gene family that encodes integrin αv, heterodimerizes with the integrin β3 chain that leads to improvement of angiogenesis and embryo attachment. Accordingly, deregulation of *ITGAV* gene has been reported in a variety of reproductive disorders suggesting its essential role in the human reproduction processes. It is reported that the expression of αvβ3 integrin, a cell surface adhesion molecule, was elevated during implantation in humans and its endometrial expression was reduced in infertile women suggesting that it is important for the process of implantation (30).

The last gene, *LIF*, encodes a multi-functional cytokine that plays a key role in the implantation process. *LIF*, belongs to the IL-6 family and plays a clear role in the process of implantation by regulating a variety of biological processes during blastocyst implantation (31). The absence of *Lif* in mice reduces blastocyst implantation. A recent study by Shokrzadeh et al. (32) indicated that administration of calcitonin during the implantation window improves endometrial receptivity in mice by up-regulating *LIF* and Le‐7a miRNAs and down-regulating that of Muc‐1. Increased expression of endometrial *LIF* in the secretory phase is essential for implantation in humans, because multiple critical events during implantation are regulated by *LIF* such as promoting the endometrial receptive state, endometrialembryo interaction, decidualization of stromal cells, and development of the blastocyst. In addition, leukemia inhibitory factor-mediated adhesion molecules require integrin αvβ3 and αvβ5 for the adhesion of trophoblast cells to endometrial cells. Gremlich et al. (33) showed in their study that IVF patients with lower plasma concentrations of *LIF* had an increased risk of implantation failure.

Most previous studies have only investigated the biochemical compounds of FF and its relation with the quality of the ovum. In contrast, the present study investigated the effect of FF on endometrial cells and it was observed that FF induces the expression of the genes that are involved in Embryo implantation. FF is rich in steroidal hormones (34), especially estrogen and progesterone, and previous studies have shown that the expression level of *HOXA10* and *HOXA11* in endometrial cells are upregulated by these hormones (35). It seems that these steroidal hormones have induced the expression of the *HOX* genes that, in turn, have functioned as transcription factors leading to the upregulation of *LIF* in the endometrial cells. Many related studies have suggested *LIF* as a positive regulator of integrins αv and β3. Therefore, *LIF* is a key regulator of the fetus implantation, as it has in turn induced the expression of the adhesion molecules *ITGAV* and *ITGB3* (36).

Unlike the study of Hashish et al. (25) that used FF from a single mature oocyte, we used pooled FF obtained from 15 mature follicles to treat the endometrial stromal cells. Our results showed a higher expression level of endometrial receptivity genes suggesting that FF can be an inexpensive, more readily available source of cytokine and growth factors and may be applicable for improving implantation rates in ART cycles.

In case of SP, unlike with FF, we only observed a significant up-regulation in the level of *LIF* gene. Considering the fact that the cytokine LIF has multiple roles in the implantation process and functions as a mediator in some other pathways (37), it seems that it may be necessary to study other *LIF*-related genes. Many studies have indicated the positive effect of SP in the implantation process (23, 38-40) . Therefore, it is necessary to perform systematic studies to investigate the genes that are affected by SP to decipher the underlying molecular mechanisms.

## Conclusion

Taken together, our results provide evidence that SP and FF may contribute to the regulation of endometrial function by upregulating endometrial receptivity genes in human endometrial cells. Accordingly, the application of SP and FF can be considered a potential natural supplement to IVF. More research is required to evaluate the clinical importance of SP and FF in the rate of embryo implantation such as intravaginal or intracervical uses of SP and FF in IVF therapies.
